# The role of fatty acid metabolism in acute lung injury: a special focus on immunometabolism

**DOI:** 10.1007/s00018-024-05131-4

**Published:** 2024-03-08

**Authors:** Xiao Lu, Guorui Li, Yi Liu, Guoqing Luo, Song Ding, Tianyu Zhang, Ning Li, Qing Geng

**Affiliations:** Department of Thoracic Surgery, Renmin Hospital of Wuhan University, Jiefang Road 238, Wuhan, 430060 China

**Keywords:** Acute lung injury, Fatty acid metabolism, Metabolic reprogram, Obesity

## Abstract

Reputable evidence from multiple studies suggests that excessive and uncontrolled inflammation plays an indispensable role in mediating, amplifying, and protracting acute lung injury (ALI). Traditionally, immunity and energy metabolism are regarded as separate functions regulated by distinct mechanisms, but recently, more and more evidence show that immunity and energy metabolism exhibit a strong interaction which has given rise to an emerging field of immunometabolism. Mammalian lungs are organs with active fatty acid metabolism, however, during ALI, inflammation and oxidative stress lead to a series metabolic reprogramming such as impaired fatty acid oxidation, increased expression of proteins involved in fatty acid uptake and transport, enhanced synthesis of fatty acids, and accumulation of lipid droplets. In addition, obesity represents a significant risk factor for ALI/ARDS. Thus, we have further elucidated the mechanisms of obesity exacerbating ALI from the perspective of fatty acid metabolism. To sum up, this paper presents a systematical review of the relationship between extensive fatty acid metabolic pathways and acute lung injury and summarizes recent advances in understanding the involvement of fatty acid metabolism-related pathways in ALI. We hold an optimistic believe that targeting fatty acid metabolism pathway is a promising lung protection strategy, but the specific regulatory mechanisms are way too complex, necessitating further extensive and in-depth investigations in future studies.

## Introduction

Acute lung injury (ALI) and its most severe manifestation, acute respiratory distress syndrome (ARDS), are relatively common, costly and potentially fatal diseases resulting from a range of intra- and extra-pulmonary factors, including sepsis, surgery, trauma, ischemia–reperfusion, pneumonia, drug toxicity, and mechanical ventilation [1, 2]. The clinical manifestations of ALI are mainly characterized by diffuse pulmonary infiltration, refractory hypoxemia and respiratory distress, while the pathological manifestations of which are the injury of pulmonary capillary endothelial cells and alveolar epithelial cells, diffuse edema of alveoli and pulmonary interstitium, severe intrapulmonary inflammation and damage of alveolar-capillary barrier [3]. Reputable evidence from multiple studies strongly suggests that excessive and uncontrolled inflammation plays an indispensable role in mediating, amplifying, and protracting lung injury. Immoderate production of pro-inflammatory cytokines and chemokines, such as TNFα, IL-1β and IL-6, can promote the recruitment and activation of inflammatory cells and activate neutrophils to secrete leukotrienes, which further aggravating lung injury [4]. Despite the improved outcome with mechanical ventilation, the period prevalence of ARDS is 10.4% of ICU admissions in 50 countries and the mortality of ARDS still remains as high as 35–55% [5, 6]. Therefore, a comprehensive elucidation of the pathophysiological mechanism of ALI is urgently warranted for better prognosis prediction and identification of potential therapeutic targets. However, due to the intricate pathogenesis of ALI, its precise mechanism remains incompletely understood.

Fatty acids (FAs) are important components of fat, cholesterol esters, and phospholipids. The metabolic process of fatty acids encompasses their cellular uptake and storage, transport to mitochondria, mitochondrial oxidation, and synthesis. Extensive attention and profound research efforts have been dedicated to comprehending the alterations of fatty acid metabolism in lung cancer. Traditionally, immunity and cellular metabolism is regarded as separate functions regulated by distinct mechanisms. Recently, more and more evidence has shown that immunity and cellular metabolism exhibited a strong interaction which has given rise to an emerging field of immunometabolism [7]. Researchers have come to realize that energy metabolism is intricately intertwined with immunoreaction and inflammation, forming the basis of the pathogenesis of numerous human diseases. Although numerous studies have confirmed alterations in various key enzymes within the fatty acid metabolism pathway in ALI/ARDS, the precise mechanism underlying the extensive changes in fatty acid metabolism and their role in the occurrence and progression of ALI/ARDS remain unclear. Currently, there is a lack of reviews on the involvement of fatty acid metabolism in ALI/ARDS. Therefore, this review aims to summarize the modifications occurring within the fatty acid metabolism pathway during ALI/ARDS and the potential lung protective strategies targeting this metabolic process.

## Main fatty acids metabolism pathways in lung

Mammalian lungs are metabolically active organs, and the dietary fat intake can modulate the composition of phospholipid fatty acids in lung membrane, thereby inducing alterations in membrane fluidity and influencing cellular functions such as hormone receptor binding and the activity of membrane-associated enzymes [8]. Cellular fatty acid metabolism encompasses a diverse array of metabolic pathways. Cells in lung can uptake FAs from extracellular sources, including lipoproteins (chylomicrons, LDL, and VLDL) processed by endonucleases, free FAs, and those obtained through macrophage phagocytosis, all of these play a crucial role in maintaining the intracellular FA pool. The intracellular sources of FAs include de novo fatty acid synthesis, lipolysis, lipophagy, and hydrolysates of glycerol phospholipids. Catalyzed by acetyl-CoA carboxylase and fatty acid synthase (FASN), the de novo synthesis of fatty acids utilizes extracellular acetate, glucose, and amino acids (including glutamine) to biosynthesize palmitates, phospholipids, triglycerides, and other fatty acids that play crucial roles in energy storage, membrane biogenesis, and signal transduction [9]. The synthesis pathway of fatty acids is intricately linked to the cellular energy state due to its substantial energy consumption. Type II alveolar epithelial cells, primarily responsible for pulmonary surfactant production, must sustain lipid synthesis even under conditions of energy stress to reduce alveolar surface tension [10]. The microenvironment of alveoli is relatively rich in lipid. When the cells are nutritionally sufficient, surplus fatty acids in the cells are esterified into inert triglycerides and gathered in the endoplasmic reticulum phospholipid bilayer to form lipid droplets (LDs) [11]. Lipid droplets are special organelles that regulate lipid balance and energy storage in the lungs. Fatty acids stored in LDs are mobilized in the form of triglyceride through lipolysis and lipophagy when needed, which is catalyzed by adipose triglyceride lipase (ATGL), hormone-sensitive lipase (HSL) and monoacylglycerol lipase (MAGL). Besides, glycerol phospholipid can be deacylated by phospholipase A and B to form lysophospholipid, and then further deacylated by lysophospholipase to form glycerophosphate and free FAs.

Free lipids play a pivotal role in cellular signal transduction and are involved in the regulation of pulmonary inflammation. For instance, eicosanoids serve as effective short-range messengers, functioning as vital biological signaling molecules. Arachidonic acid, a 20-carbon fatty acid, serves as the precursor for numerous signaling lipids. Prostaglandins are metabolites of arachidonic acid and their levels are up-regulated in many inflammatory lung injuries [12]. Generally speaking, free fatty acids (FFAs) necessitate activation into the form of fatty acyl-CoA (FA-CoA) through the catalysis of long-chain acyl-CoA synthetase. FA-CoA is a transient compound generated through the conjugation of CoA with the terminal end of long-chain acids. Subsequently, FA-CoA can undergo desaturation by stearoyl-CoA desaturase or fatty acid desaturase (FADS1, FADS2), and elongation by very long-chain fatty acid elongases to produce monounsaturated or polyunsaturated fatty acids, which can be incorporated into more complex lipids such as membrane glycerophosphatide.

The fatty acid oxidation (FAO) of alveolar epithelial cells plays a pivotal role in maintaining the functional homeostasis of pulmonary cells [13]. Previous studies have shown that β-oxidation of fatty acids in the rat lungs is up-regulated by nearly 40% during starvation, suggesting that FAO serves as a crucial source of energy for the lungs during energy depletion [14]. With each cycle of fatty acid oxidation, the FA-CoA chain is shortened by two carbon atoms until it is ultimately converted into acetyl-CoA. The peroxisomal β-oxidation of very long-chain fatty acyl-CoA (VLCFA-CoA) generates acetylcarnitine and short-chain acyl carnitines, which are the substrates of mitochondrial oxidation. Long-chain FA-CoA is transported to mitochondria through carnitine palmitoyltransferase (CPT) system consisted of CPT1, CPT2 and carnitine-acylcarnitine translocase, while short-chain and medium-chain FA-CoA passively diffuses across the membrane. Saturated fatty acyl-CoAs undergo oxidation via the combined actions of acyl-CoA dehydrogenase, enoyl-CoA hydratase, hydroxyacyl-CoA dehydrogenase, and 3-ketoacyl-CoA thase enzymes, which collectively constitute the β-oxidation pathway. On the other hand, unsaturated fatty acyl-CoAs require an additional auxiliary pathway involving Δ3, Δ2-enoyl-CoA 2,4-dienoyl CoA reductase 1 to eliminate their double bonds before they can re-enter β-oxidation. These reactions generate acetyl-CoA for the tricarboxylic acid cycle, as well as FADH2 and NADH, which act as substrates for the electron transport chain [15]. The β-oxidation pathway is a prominent adenosine triphosphate source in the lung, however, it concurrently induces more pronounced oxidative stress in pulmonary cells compared to other organs.

## Alteration of fatty acid metabolism in lung during acute lung injury

Typically, lung tissue consumes moderate energy compared with other functional tissues such as heart or skeletal muscles. However, similar to other metabolically active tissues, mitochondrial respiration accounts for 80% of ATP production in lung tissue, with the remaining 20% derived from alternative sources [16]. When challenged by the inflammation of ALI, the lung alters in metabolism and energy consumption. The model of ALI mice exhibits metabolic characteristics that are consistent with changes in energy production and reduction in ATP levels [17].

**Fig. 1:**
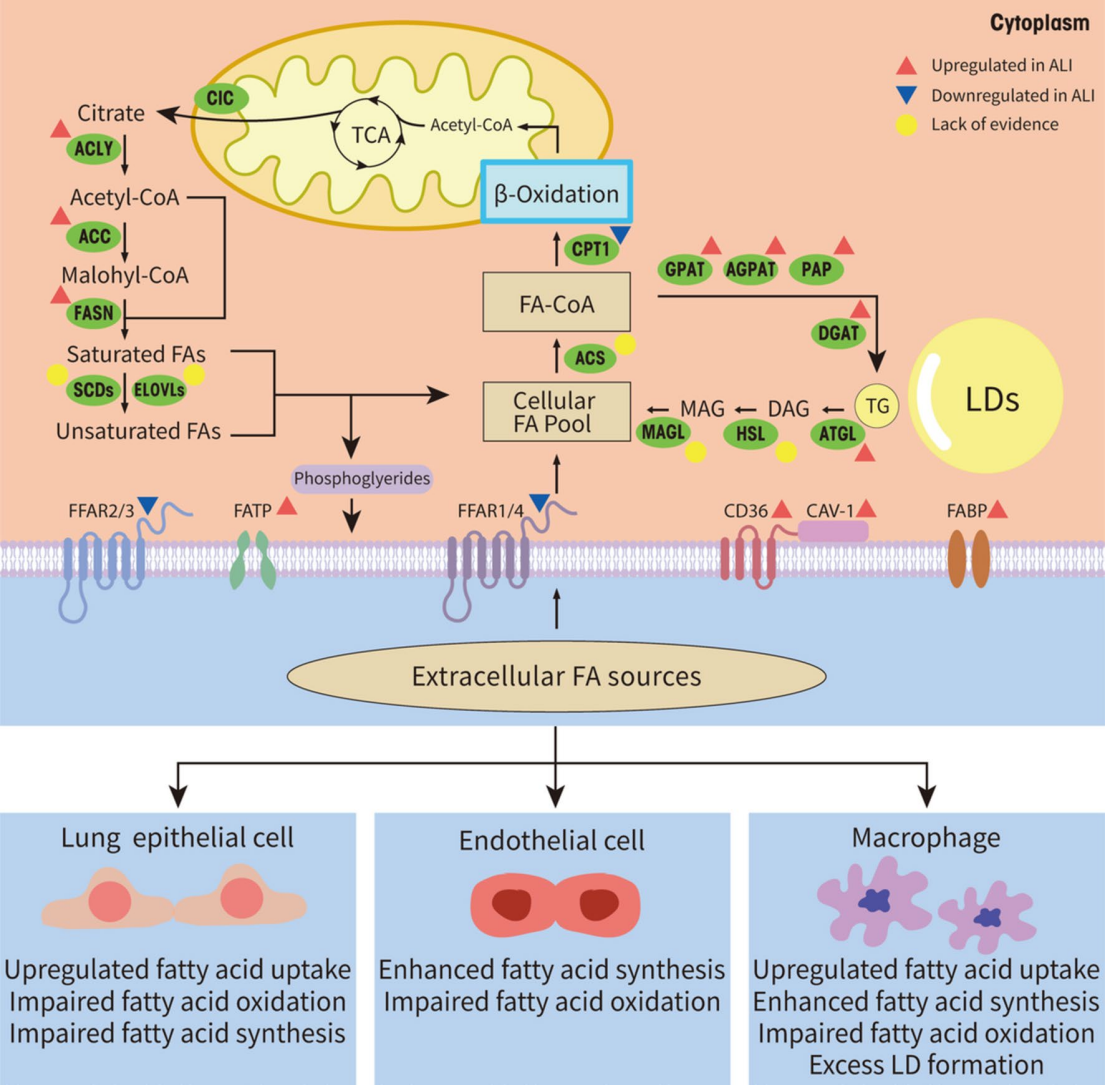
Main fatty acids metabolism pathway in lungs and alteration during acute lung injury. The metabolic process of fatty acids encompasses their cellular uptake and storage, transport to mitochondria, mitochondrial oxidation, and synthesis. Lung epithelial cells can uptake FAs from extracellular sources, including lipoproteins, free FAs, through a series of specialized transporters such as FABP, FATP, CD36, Caveolin-1 and FFAR. The intracellular sources of FAs include de novo fatty acid synthesis, lipolysis, lipophagy and hydrolysates of glycerol phospholipids. De novo fatty acid synthesis occurs in the cytoplasm, where citrate is converted to the final long-chain saturated or unsaturated FA. These steps are catalyzed by ACLY, ACC, FASN, and desaturases, as well as elongases. Subsequently, some FAs are stored in LDs in the form of triglycerides or mobilized through β-oxidation to generate energy and acetyl-CoA which returns to the TCA circle, while the other FAs are incorporated into cellular phospholipids and released from cell membranes to serve as important lipid mediators. During ALI, inflammation and oxidative stress lead to energy depletion, resulting in impaired fatty acid oxidation, increased expression of proteins involved in fatty acid uptake and transport, enhanced synthesis of fatty acids, and accumulation of LDs as well as up-regulated lipolysis

Previous research demonstrates that in the case of ALI, there is significant impairment of FAO in pulmonary epithelial cells, resulting in reduced biological activity and even apoptosis of lung epithelial cells [13]. In addition, glycolysis is up-regulated in lungs during acute inflammation as a metabolic reprogramming response typically employed to compensate for impaired mitochondrial oxidative phosphorylation activity [18]. These findings validate the role of FAO in the pathogenesis of alveolar epithelial cell (AEC) dysfunction and ALI. Sustaining mitochondrial function amidst acute lung injury is advantageous for withstanding energy stress. However, only by employing the carbon isotope tracer technique in future studies can we accurately determine the respective contributions of fatty acid and glucose oxidation to AEC bioenergy generation. On the other hand, there is an upregulation of most proteins involved in fatty acid uptake and transport in ALI, indicating an enhanced breakdown of fats to meet the increased energy demands. This may be a passive adjustment, and some studies have shown that a large number of ceramides and triglycerides are produced in the early stage of ALI [19], and FA is depleted in this process, so proteins responsible for fatty acid uptake and transport are passively up-regulated to supplement consumed FA, meanwhile, they also play a crucial role in the regulation of pulmonary glycolipid balance, secretion of inflammatory factors, generation of ROS, and release of leukotriene [19–21]. In addition, fatty acid synthesis also increases in ALI, primarily related to inflammatory signal transduction and regulation of lipid biosynthesis in immune cells such as macrophages. On one hand, the proteins mediating the de novo synthesis of fatty acid promote the secretion of pro-inflammatory cytokines such as ROS, IL-6 and IL-1β, on the other hand, they are crucial for maintaining cell function under the challenge of acute lung injury. As mentioned, the metabolic reprogramming during ALI is characterized by enhanced fatty acid uptake, suppression of fatty acid oxidation and oxidative phosphorylation, as well as fragmentation of tricarboxylic acid (TCA) cycle, all of which collectively create an environment conducive to triglyceride synthesis in inflammatory macrophages, thereby facilitating lipid droplet formation [22]. LDs can serve as precursors for the biosynthesis of the pro-inflammatory mediator prostaglandin E2 (PGE2). Moreover, the inflammatory activation of vascular endothelial cells is a common consequence of acute lung injury caused by different pathogenic mechanisms, which can further promote the formation of LDs. In addition to the key enzymes involved in fatty acid metabolism, numerous FFAs and their derivatives also participate in the pathogenesis of acute lung injury and exhibit diverse functionalities. Overall, the alterations in the pathway of fatty acid metabolism during acute lung injury are intricate, and changes in individual molecules or protein expressions cannot be solely categorized as beneficial or unbeneficial, rather, they involve a complex metabolic regulatory network that warrants further profound exploration (Fig. 1).

## The impact of free fatty acids uptake and transportation on ALI

The fatty acids play a crucial role in cellular energy production and serve as precursors for all types of lipoids, including those involved in the formation of biofilms. As a potent signaling molecule, free FAs regulate many cellular processes related to lipid metabolism and the development of metabolic syndrome. Furthermore, free FAs can also act as endogenous ligands for specific receptors, including Toll-like receptors (TLR) 2 and TLR4[23]. Normally, tissue cells prioritize the uptake of exogenous FAs from the microenvironment to support biosynthesis and energy production, which depends on specialized transporters such as fatty acid-binding proteins (FABP), family of fatty acid transport protein (FATP), CD36, Caveolin-1 and free fatty acid receptor (FFAR)[24]. Different fatty acid transporters and receptors exhibit distinct pro-inflammatory and anti-inflammatory properties in the case of acute lung injury, which involves quite complex regulatory mechanisms.

### Fatty acid-binding proteins

FABP, a group of low molecular weight proteins consisting of 12 family members, functions as a lipid chaperone that facilitates the transport of fatty acids to various organelles including mitochondria, peroxisomes and nucleus. FABP4, also known as adipocyte protein 2 or aP2, is a cytoplasmic lipid binding chaperone protein with a molecular weight of approximately 15 kDa mainly expressed in the lung. Notably, it is mainly expressed in adipocytes, macrophages and endothelial cell subsets [25]. According to recent research, there is an elevation of serum FABP4 levels during early sepsis-induced inflammation, primarily arising from the response of adipocytes to accelerated fat decomposition signals [26], which is associated with an increased risk of impaired lung function [27]. Elevated FABP4 can aggravate the severity of inflammation by promoting the release of pro-inflammatory cytokines such as TNF-α and IL-6, which is partly regulated by vasorin (VASN). VASN, a glycoprotein encoded by the VASN gene and located on the plasma membrane, serves as an upstream regulator of FABP4. VASN deficiency leads to an upregulation of FABP4, subsequently resulting in the elevation of IL-1β, TNF-α and IL-6, thereby exacerbating lung injury [28]. By establishing cecal ligation and puncture (CLP)-induced ALI murine model and lipopolysaccharide (LPS)-induced A549 cell model, Gong et al. [29] demonstrate that inhibition of FABP4 can effectively suppress ROS expression, thereby reducing the production of inflammatory cytokines such as C-X-C motif chemokine ligand 8 (CXCL-8), TNFα, IL-6, and IL-1β both in vivo and in vitro, providing a novel insight into the protective role of blocking FABP4 in LPS-induced ALI. Moreover, inhibition of FABP4 can alleviate hyperoxia-induced lung injury and pulmonary fibrosis in neonatal rats via inhibiting TGF-β signal transduction [30]. However, in the mouse model of Pseudomonas aeruginosa pneumonia, FABP4^−/−^ mice exhibit decreased bacterial clearance rate, delayed neutrophil recruitment, exacerbated lung injury, and increased mortality compared to wild type (WT) mice after nasal infection with Pseudomonas aeruginosa. This study finds that macrophage-derived FABP4 can promote host defense by regulating the production of CXCL-1, indicating that FABP4 plays a pivotal role in promoting host immune defense [31]. In conclusion, FABP4 is a promising therapeutic target for ALI.

Recent studies have demonstrated that FABP5 exerts a protective role in pulmonary bacterial infection and smoking-induced pulmonary inflammation through upregulation of PPARγ activity [32, 33]. Similarly, in the influenza A H1N1 infection model, FABP5^−/−^ mice show increased inflammatory cells and more severe lung injury [34]. The importance of protein S glutathione phosphorylation and Grx1 has been studied in many inflammatory lung diseases. FABP5 is the main target of Grx1 and is easily regulated by redox dependence in oxidative stress. Deletion of the macrophage-specific Grx1 gene exhibits a protective effect against acute lung injury in mice. S-glutathionylation promotes fatty acid-binding and nuclear translocation of FABP5, activates PPARβ/δ, and suppresses macrophage inflammation. S-glutathionylation of FABP5 acts as an anti-inflammatory factor in response to oxidative stress during acute lung injury, providing a potential therapeutic target for its treatment [35].

In the lung, epidermal FABP (E-FABP) is involved in the synthesis of dipalmitoyl phosphatidylcholine, the main component of alveolar surfactant, thus, all the type II AECs (AECIIs) are capable of expressing E-FABP [36]. However, the impairment of the surfactant system is observed in ALI. Veronika et al. [37] found that exogenous keratinocyte growth factor (KGF), a protein playing important roles in lung development, inflammation and repair, significantly induces the proliferation of AECIIs and up-regulate the expression of E-FABP gene and protein in the whole lung, suggesting a potential relation to the molecular mechanism underlying KGF-mediated lung protection. Enhanced E-FABP may contribute to lung protection by up-regulating surfactant synthesis, enhancing resistance against oxidative stress, and stabilizing leukotriene A_4_.

### Family of fatty acid transport proteins

The FATP family proteins, also officially named as solute carrier family 27 (SLC27), play a pivotal role in the transport and activation of fatty acids. FATP is present in the cell membrane and organelles, exhibiting FA-CoA ligase activity. The mammalian FATP family comprises six members, each showing distinct expression patterns. They demonstrate varying degrees of specificity in the transport or activation of fatty acids and are associated with lipid disorders. In order to assess the role of FATP in macrophages, Nishiyama et al. [19] established a RAW264.7 cell model stably expressing FATP1, FATP4 and FATP6. Their quantitative real-time PCR (qPCR) results showed that FATP1 and FATP4 increased significantly in M1 macrophages, while M2 macrophages exhibited a marked increase in FATP6 expression. There is no significant difference in the expression of FATP2, FATP3 and FATP5 among M1 and M2 macrophages as well as unstimulated macrophages. FATP1 is the most highly expressed isomer in macrophages, suggesting that FATP1 is mainly involved in the uptake of FAs by macrophages. The overexpression of FATP1 enhances cytokine production of macrophages, whereas the overexpression of FATP4 and FATP6 does not exert the same effect. This functional disparity can be attributed to the distinct fatty acid transported by different FATP. It is known that the substrates of FATP1 are C16:0, C18:1, and C24:0, while the substrate of FATP4 is C24:0, and substrates of FATP6 are C18:1, C20:4, and C24:0 [38]. Therefore, FATP1-transported C16:0 plays a key role in the production of inflammatory cytokines. C16:0 activates TLRs and inflammasomes, thereby leading to the production of pro-inflammatory cytokines such as TNFα, IL-6 and IL-1β [39], while FATP1 contributes to the aforementioned process by uptaking C16:0. Interestingly, in the absence of LPS induction, overexpression of FATP1 did not result in an increase in macrophage FA uptake and ceramide levels, suggesting the potential mechanism may be that LPS initially stimulating ceramide and triglyceride synthesis, in which FA is consumed in cells, and then promotes macrophages to absorb FA through FATP1 to recover the consumed FA. Furthermore, Nishiyama et al. also found that FATP1 can enhance macrophage inflammatory response by coupling with ceramide and c-Jun N-terminal kinase signals [19]. These findings suggest that targeted inhibition of FATP1 could serve as a promising therapeutic approach for suppressing the production of inflammatory cytokines and restraining lung-resident macrophage activity.

### CD36

CD36, also known as fatty acid transferase, is a member of the class B scavenger receptor family mainly involved in lipid uptake, immune recognition and cell adhesion. CD36 can recognize many ligands and is widely expressed on the surface of various cell types, including macrophages and endothelial cells. It is noteworthy that the specific knockout of CD36 in endothelial cells results in an elevation of circulating FA levels and a reduction in FAs uptake in the heart, skeletal muscle, and brown adipose tissue; however, this decrease is not observed in the liver, kidney, and lung, indicating that CD36-mediated fatty acid uptake by endothelial cells may not be the main regulator of fatty acid uptake in liver, kidney and lung [40]. Interestingly, however, CD36 still plays an important role in mediating diverse forms of lung injury. CD36 functions as a ligand for truncated oxidized phospholipids (Tr-OxPLs), which are generated through the oxidation of cell membranes or circulating lipoproteins. Intriguingly, both Tr-OxPLs and CD36 play crucial roles in endothelial dysfunction-derived acute lung injury. Notably, CD36-knockout mice and those treated with a synthetic amphipathic helical peptide L37pA targeting CD36 exhibited enhanced resistance against Tr-OxPL-induced lung injury, underscoring the indispensable involvement of CD36 in mediating Tr-OxPL-induced endothelial cell dysfunction and suggesting significant therapeutic potential of CD36 inhibitory peptides in mitigating lung injury and inflammation [41]. On the other hand, CD36 expressed on alveolar macrophages (AMs) mainly mediates the phagocytosis of anionic phospholipids, fatty acids and albumin by AMs, which is essential for maintaining the structure and functional homeostasis of the lung. During ALI inflammation, in sharp contrast to M1 macrophages, M2 macrophages are characterized by a complete TCA cycle and enhanced mitochondrial oxidative phosphorylation (OXPHOS) to maintain sustained energy production [42]. The M2 macrophages internalize the fatty acids through CD36 and utilize them for energy production via FAO, thereby providing fuel for OXPHOS. According to a recent research, CD36 in macrophages plays a critical role in the pathogenesis of ALI by regulating macrophage M1 polarization. In terms of the underlying mechanism involved, CD36 promotes the association between CD14 and TLR4, leading to NF-κB activation and facilitating LPS-induced M1 polarization [21]. Besides, synthetic amphipathic helical peptides (SAHPs), which also exhibits relative selectivity towards CD36, exert a significant impact on inhibiting acute pulmonary inflammation and dysfunction, and can be regarded as a potential novel approach for treating ALI and pulmonary edema [43].

### Caveolin-1

Caveolin-1 (CAV-1) is involved in a variety of physiological processes, including transmembrane transport, endocytosis, lipid metabolism and signal transduction, and it is expressed in almost all types of cells within the lung, including type I epithelial cells, endothelial cells, smooth muscle cells, fibroblasts, macrophages and neutrophils, and plays an important role in the pathogenesis of acute lung injury [44].

Activation of CAV-1/NF-κB axis is related to the inflammation induced by LPS and autophagy in ALI [45]. The regulation of multiple inflammatory mediators mediated by NF-κB is essential for the pathogenesis and progression of ALI inflammation, more importantly, CAV-1 plays an intermediary role in many signal pathways that lead to the activation of NF-κB, and the expression levels of CAV-1 and NF-κB are significantly up-regulated during ALI [46]. Knockout of the CAV-1 can effectively inhibit NF-κB activation and inflammatory cell infiltration, thereby significantly reducing overall mortality in LPS-induced ALI mice [20]. On the other hand, suppressing the expression of CAV-1/ NF-κB axis can significantly ameliorate ALI by activating autophagy-related AMP-activated protein kinase (AMPK), restraining AKT/mTOR pathway activation, and attenuating inflammatory response in CD4/80^+^ macrophages and CD3^+^ T lymphocytes [45], suggesting that CAV-1/ NF-κB axis is a potential therapeutic target for ALI.

Interestingly, however, studies have shown that up-regulating CAV-1 can also protect against ALI. Dexmedetomidine is a highly potent and selective α2-adrenoceptor agonist, apart from its well-established sedative and analgesic properties, it has been shown to ameliorate septic ALI by inhibiting the release of pro-inflammatory cytokines mediated by TLR4/ NF-κB signaling pathway. During this process, the downstream molecule CAV-1 that regulates TLR4MEDIATED inflammation, is up-regulated and inhibits the activation of TLR4/ NF-κB signal pathway [47]. Silencing information regulator 1 (SIRT1) is involved in mediating the anti-inflammatory effect after lung injury. The activation of SIRT1 reduces the secretion of pro-inflammatory cytokines and alleviates endotoxin-induced inflammatory response in lungs, whose regulatory mechanism is related to the deacetylation of signal transducer and activator of transcription 3 (STAT3). SIRT1 inhibits the activity of STAT3 and ultimately inhibits the transcription of inflammation-related genes. Cav-1 is a downstream target gene of SIRT1, and their combination exhibits a synergistic effect, while Cav-1 also serves as a downstream gene targeting STAT3 and directly interacts with it on the lipid raft [48]. In the endotoxin-induced ALI model, the expression of SIRT1 and CAV-1 was suppressed, leading to an overexpression of STAT3, TLR4, TNF-α, and IL-6. Upon administration of SIRT1 agonist, the up-regulated SIRT1 enhanced Cav-1 protein and mRNA expression by down-regulating STAT3 and antagonizing its injurious effects. Consequently, the lung injury was alleviated. The presence of SIRT1 agonist facilitated a significant anti-inflammatory effect through the promotion of Cav-1 via the SIRT1/STAT3 complex. These findings provide evidence for synergistic lung protective effects mediated by the SIRT1/STAT3 axis and Cav-1 [49].

### Free fatty acid receptor

FFAR belongs to the G protein-coupled receptor family. At present, the extensively studied FFARs mainly include FFAR2(GPR43), FFAR3(GPR41), FFAR1(GPR40), FFAR4(GPR120). The former two can be activated by short-chain FFAs, while the latter two are mainly activated by medium- and long-chain FFAs. As the endogenous ligands of FFARs, FFAs activate the FFARs by binding to their corresponding extracellular components of FFAR, dissociating the α subunit of the intracellular coupled heterotrimer G protein from the βγ subunit, thus further affecting the intracellular signal proteins involved in various cellular physiological and pathological processes and exerting their functions [50].

FFAR2 is highly expressed in different tissues and cells, especially in immune cells such as neutrophils, eosinophils and monocytes. FFAR2 is involved in the regulation of appetite and secretion of gastrointestinal peptides, thus regulating the decomposition and formation of fat. Meanwhile, FFAR2 also acts as a crucial receptor for acetate and plays an indispensable role in the "gut-lung axis" by serving as a sensor of the host gut microbiota activity through binding with acetate to diminish susceptibility to pathogens and facilitates an appropriate immune response within the lungs [51]. In general, FFAR2 exhibits anti-inflammatory and antioxidant stress effects in ALI, and the expression of FFAR2 is related to the increase of 30-day survival in patients with sepsis [52]. Up-regulated FFAR2 is involved in the inactivation of NLRP3 inflammasomes in septic macrophages by regulating mitochondrial fission, thereby mitigating ROS-induced mitochondrial damage and suppressing inflammation [53]. In another study, Xu et al. [54] find that FFAR2 expression is significantly decreased in LPS-induced ALI mice and AECII cell models, while overexpression of FFAR2 can ameliorate LPS-induced lung injury and apoptosis through JNK/ELK1 signaling pathway, which further confirms the protective role of FFAR2 in ALI. Baicalin, a flavonoid derived from Scutellaria baicalensis Georgi, has been found by Peng et al. to possess the ability to modulate dysbiosis of gut microbiota induced by avian pathogenic Escherichia coli (APEC). This modulation leads to an increase in the production of short-chain fatty acids (SCFAs), particularly acetic acid, within the gut. Subsequently, the elevated levels of acetate may circulate to the lungs and activate FFAR2 as a defense mechanism against APEC infection [55].

FFAR2 and FFAR3 differ in their affinity for SCFAs. FFAR2 has a similar affinity for acetate, propionate, and butyrate, whereas FFAR3 has greater affinity for propionate than butyrate and low affinity for acetate [56]. SCFAs are by-products of dietary fiber metabolism of gut microbiota, whose anti-inflammatory properties in ALI/ARDS have been proved in various studies. Acetate can ameliorate ischemia–reperfusion (IR)-induced acute lung inflammation via the FFAR2/FFAR3 signaling pathway [57].

Functional FFAR1 expresses highly in human lungs [58], but how FFAR1 affects ALI still remains unclear. FFAR4 was strongly expressed in pulmonary epithelial cells, playing a role in initiation of innate immunity, cellular protection, and immune cell migration within the pulmonary system [59]. It is also reported that ω-3 polyunsaturated fatty acids can accelerate airway repair by activating FFAR4 in club cells, which may provide supporting evidence for future clinical trials of ω-3 fatty acids in subjects with acute airway injury [60]. Another gut microbe-induced ω-3 fatty acid 18-hydroxy eicosapentaenoic acid (18-HEPE) can partially enhance IFN-γ production through FFAR4, thereby increasing influenza virus infection resistance [61].

Finally, GPR84, a medium-chain fatty acid receptor, plays a key role in the occurrence and development of ALI by regulating the function of neutrophils. GPR84 was highly up-regulated in cells isolated from bronchoalveolar lavage fluid (BALF) of LPS-induced ALI mice. Further studies found that the activation of GPR84 strongly induced neutrophils to produce ROS by stimulating the activation of Lyn, AKT and ERK1/2 and the assembly of NADPH oxidase. The lack or blockage of GPR84 can significantly improve the pulmonary inflammation of ALI mice by reducing neutrophil infiltration and oxidative stress [62].

## The impact of fatty acid oxidation on ALI

Alveolar epithelial cells undergo a large amount of FAO under normal physiological conditions [13], however, this metabolic process is impaired during ALI. The pathogenesis of ALI involves the activation of *N*-methyl-D-aspartate receptor (NMDAR) [63], simultaneously, NMDAR activation disrupts FAO by attenuating the phosphorylation and activity of PPARα through the ELK1/2 pathway, thereby promoting the lipid accumulation induced by palmitic acid [64].Overproduced ROS during ALI also results in the suppress of the expression of PPARα and CPT1A [65], while impaired FAO is also found to be associated with RIPK3-mediated necrotizing apoptosis [66]. Interestingly, however, Cui et al. [13] observe a significant decrease in FAO and down-regulation of key regulators of FAO and mitochondrial bioenergy generation including PGC-1α, CPT1a, MCAD and LCAD when treating mouse alveolar epithelial cell line MLE-12 cells with ALI mouse BALF. Same result is obtained when MLE-12 cells are exposed to the pro-inflammatory cytokine TNFα. These findings suggest that FAO impairment in AECs is the secondary effect of pro-inflammatory cytokines in ALI. Further investigation into the mechanisms by which pro-inflammatory signals inhibit the expression of key mediators in FAO help distinguish pulmonary inflammation from AEC dysfunction, so as to provide novel therapeutic strategies for treating ALI. Currently, the protective effect of up-regulating FAO expression and its key mediators on pulmonary endothelial cells has also been validated in an ALI murine model induced by hyperoxia [67]. Both L-carnitine and baicalin can mitigate the dysregulation of apoptosis, migration, and angiogenesis in pulmonary endothelial cells induced by hyperoxia through upregulation of CPT1A [68].

During sepsis-induced severe systemic inflammatory response syndrome, the expression of CPT1A is downregulated, leading to a reduction in FAO. Consequently, plasma non-esterified fatty acid (NEFA) levels increase while energy supply to corresponding organs decreases [69], ultimately resulting in the accumulation of lipids in the body. Several types of NEFAs exert cytotoxic effects by activating Toll-like receptors and inhibiting Na^+^/K^+^-ATPase, thereby exacerbating to lung injury. Studies have demonstrated that mediterranean diet rich in olive oil can mitigate the occurrence of various diseases and induce down-regulation of circulating inflammatory biomarkers and oxidative stress [70]. The main component of olive oil is omega-9 oleic acid (OA), a MUFA. Cassiano et al. [71] find that oleic acid treatment of septic mice can up-regulate the expression of CPT1A and uncoupling protein 2 (UCP2) through AMPK/PPAR pathway, thereby promoting FAO, reducing plasma NEFA levels, ameliorating organ damage, and enhancing survival rates in septic mice.

The level of acyl-CoA in serum and plasma serves as a more intuitive index for reflecting FAO, however, due to the analytical challenges for measuring acyl-CoA content in serum and plasma, acylcarnitine remains the optimal alternative measure for evaluating FAO. In sepsis, an elevation in plasma acetylcarnitine level is observed and found to be associated with increased mortality [72]. Furthermore, patients with high inflammatory subtypes exhibit a significant increase in acetylcarnitine and the ratio of acetylcarnitine to carnitine, indicating more severe metabolic disturbances in high inflammatory subtypes [73]. Moreover, In ALI caused by renal ischemia–reperfusion injury, the alteration of FAO pathway is the most significant, which is characterized by an elevation in medium-chain and long-chain acylcarnitine levels, suggesting a systemic impairment of mitochondrial function across various organs and cell types, including the lungs [74]. Mitochondria serve as the most important sites for FAO. In a case–control autopsy study, exceedingly elevated levels of mitochondrial oxidative damage are observed in AECIIs and AMs of patients with ARDS, when compared to individuals who die of non-pulmonary causes [75]. Besides, what related to the alteration in CPT1A expression during ALI is that inflammatory macrophages augment the reservoir of long-chain acylcarnitine, reflecting increased substrate levels due to enhanced absorption and synthesis of FAs, as well as subsequent accumulation of acylcarnitine owing to impaired FAO [22]. Non-targeted metabolomic studies conducted in septic cohorts with ARDS reveal that patients in high inflammatory subtypes exhibit decreased blood lipid levels and up-regulated glycolysis compared to those in low inflammatory subtypes [76], which is consistent with lipid metabolic disorders, namely damaged FAO and accumulation of long-chain acylcarnitines, disrupting surfactant function and exacerbating the severity of ALI [77]. The aforementioned findings suggest that mitochondrial metabolic dysfunction may hold prognostic significance in critical diseases associated with acute respiratory failure, irrespective of etiology. Suber et al. demonstrate that increased levels of acetylcarnitine, octanoylcarnitine, and 3-methylhistidine distinguish patients exhibiting high or low inflammatory subtypes of ARDS, and octanoylcarnitine and 3-methylhistidine are associated with poor outcomes [73]. This provides an additional phenotypic framework for identifying ARDS patients who could potentially benefit from targeted therapy aimed at replenishing energy reserves during periods of critical illness.

## The impact of fatty acid storage and release on ALI

When cellular nutrition is adequate, surplus fatty acids within the cell undergo esterification to form inert triacylglycerol (TG) and accumulate in the phospholipid bilayer of the endoplasmic reticulum (ER). Upon surpassing a certain limit, these lipids are released from the ER via budding mechanisms to generate LDs. De novo synthesis of TG is catalyzed by four consecutive enzymes: glycerol phosphate acyltransferase (GPAT), acylglycerolphosphate acyltransferase (AGPAT), phosphatidic acid phosphatase (PAP) and diacylglycerol acyltransferase (DGAT) [78]. The mammalian genome encodes two DGAT enzymes, namely DGAT1 and DGAT2. Lipid droplets are regarded as specialized intracellular organelles that primarily function in maintaining intracellular lipid homeostasis and energy storage. Under energy stress, fatty acids are released mainly by lipid droplet lipolysis, wherein triacylglycerol is hydrolyzed to fatty acids and glycerol by ATGL, HSL, and MAGL [79]. With the advancement of research, people gradually realize that LDs serve not only as organelles for lipid storage but also play a crucial role in mediating cellular processes such as protein binding and inactivation, fat transport, and intracellular signal transmission [80].

Alveolar macrophages (AMs) reside in the alveoli and maintain the stability of the pulmonary environment by playing a key role in immune surveillance and lipid surfactant catabolism. Lipids in alveoli mainly exist in the form of surfactants, and active lipid metabolism is essential for AMs to uphold the dynamic equilibrium of pulmonary surfactants and stabilize the local microenvironment, with CD44 primarily mediating this function [81]. CD44 deficiency disrupts lipid metabolism homeostasis in AMs, leading to accumulation of pulmonary surfactant lipids and upregulation of lipid scavenger receptor CD36, ultimately resulting in increased intracellular lipid droplet formation and exacerbation of oxidized lipid-induced lung injury [81]. Compared with other tissue-resident macrophages, AMs contain a large number of LDs and express genes related to lipid metabolism, such as peroxisome proliferators-activated receptors (PPAR), including PPARγ and PPARα [82]. Inhibition of PPARγ in normal AMs enhances lipid droplet formation [81], while activation of PPARα facilitates lipid metabolism in AMs [83]. On the other hand, AMs also specifically express the receptor Lepr, which encodes the pivotal metabolic hormone leptin, and the inherent Lepr signal of AMs can ameliorate acute lung injury. The Lepr signal maintains adenosine monophosphate-activated protein kinase activation in a calcium influx-dependent manner and restores cellular metabolism, thereby preventing excessive lipid droplet formation and alleviating metabolic stress in the fat-rich alveolar microenvironment [84]. The alterations in mitochondrial function of inflammatory macrophages during acute lung injury are characterized by changes in core metabolism, including increased fatty acid uptake, inhibition of fatty acid oxidation and oxidative phosphorylation, and fragmentation of the TCA cycle to support the production of citrate and aconitic acid for fatty acid and itaconic acid synthesis, respectively. These events collectively create an environment that promotes triglyceride synthesis, which subsequently leads to lipid droplet formation [22]. The upregulation of DGAT1 expression induced by LPS plays the leading role in the augmentation of TG synthesis, while DGAT2 merely assumes an ancillary function. Simultaneously, LDs abundant in triglyceride and cholesterol ester also serve as ample precursors for PGE2 production. Conversely, in DGAT1 deficient macrophages, the inhibition of TG synthesis results in inadequate fat storage within LDs, thereby reducing the pool of precursors responsible for prostaglandin generation and attenuating the inflammatory response of macrophages [22]. Another study demonstrates that LPS-induced activation significantly enhances triglyceride accumulation in macrophages, attributed to the upregulation of hypoxia-inducible lipid droplet-associated (HILPDA) protein. HILPDA suppresses triglyceride hydrolysis by promoting ATGL proteasomal degradation, thereby attenuating the inflammatory response of macrophages and reducing the production of PGE2 and IL-6, while deficiency of HILPDA in macrophages increases ATGL-mediated lipolysis, potentially augmenting fatty acid availability as precursors for inflammation [85].

When it comes to lung injury caused by pathogens, although LDs can facilitate the growth and reproduction of invasive pathogens by providing substrates, they also function as an initial line of defense within cells and act as a molecular switch in innate immunity, reprogramming cell metabolism and triggering protein-mediated antibacterial mechanisms [86]. As a response to LPS, a diverse array of host defense proteins, including interferon-induced guanosine triphosphatase and antibiotics, assemble into intricate clusters on LDs. Furthermore, LPS also induces the physical and functional uncoupling between LDs and mitochondria, leading to reduced fatty acid metabolism while enhancing the interaction between LDs and bacteria. Thus, LDs actively participate in mammalian innate immunity at two levels: serving as autonomous organelles that orchestrate immune protein-mediated elimination of intracellular pathogens and playing a pivotal role in regulating local and systemic metabolic adaptation during infection [87]. During SARS-CoV-2 infection-induced lung injury, the expression of lipid metabolism-related gene SREBP-1, nuclear receptor PPARγ and its downstream gene CD36 was up-regulated, leading to cellular reprogramming towards a lipogenic phenotype. This metabolic shift facilitates the synthesis of DGAT-1-dependent LDs, thereby promoting SARS-CoV-2 replication and inflammatory mediator production [88], suggesting that LDs play an important role in SARS-CoV-2 replication and regulation of immune response, and similar effects have been reported in other RNA viruses [89, 90]. Interestingly, from the middle stage of infection, LDs catabolism dependent on ATGL and HSL emerges as a common characteristic in the replication process of various RNA viruses, including SARS-CoV-2, serving as the primary source of FFAs. The viral-induced cellular damage disrupts the membrane transporter system, leading to intracellular malnutrition in later stages of infection. Consequently, those viruses increasingly rely on FFA derived from LD storage for both morphogenesis and energy generation. However, lipase inhibitor treatment which prevents LD lipolysis significantly inhibits the replication of RNA virus and the production of pro-inflammatory factors as well as reducing FAO and the palmitoylation of virus protein [91]. The inhibition of LD lipolysis, leading to a reduction in FFA release, may ameliorate the cytokine storm induced by viruses through the blockade of the interaction between FFA-driven eicosanoid storm and pattern recognition receptors (PRRs) and pathogen-associated molecular patterns (PAMPs) related to virus replication [92, 93]. These findings indicate that targeting LD-related lipase could be a potential therapeutic strategy for lung injury caused by RNA virus infections.

## The impact of fatty acid synthesis on ALI

The de novo fatty acid synthesis pathway tightly links glucose and fat metabolism, facilitating cellular adaptation to environmental changes and generating substantial ATP through β-oxidation. The substrate of fatty acid synthesis is acetyl-CoA catalyzed by ATP- citrate lyase (ACLY). Acetyl-CoA produces palmitic acid by carboxylation of acetyl-CoA carboxylase (ACC) and condensation of FASN, and the latter further forms fatty acids with different carbon chain length and saturation participating in different biological processes such as plasma membrane synthesis and signal transduction under the action of elongase of very long-chain fatty acids (ELOVL) and stearoyl-CoA desaturase 1 (SCD1) [94].

### ACLY

ACLY is considered to be the key enzyme linking glucose, glutamine metabolism and fatty acid production by decomposing citric acid to acetyl-CoA in the cytoplasm. Normally, citric acid is synthesized in mitochondria and transported to the cell membrane through mitochondrial citrate carrier (CIC), generating acetyl-CoA and oxaloacetic acid under the action of ACLY [95].

During acrolein-induced acute lung injury, ACLY is downregulated while another research demonstrates that ACLY is up-regulated in the middle-late stages of zinc chloride smoke inhalation-induced lung injury repair [96, 97]. Although there is limited direct research on ACLY and ALI, extensive investigations have been conducted regarding the alterations of ACLY in inflammatory macrophages, which may have an impact on ALI as it has been proved that macrophages are involved in the pathogenesis and progression of acute lung injury. Lipid biosynthesis is essential for membrane remodeling of M1 macrophages and synthesis of inflammatory mediators. In LPS-induced macrophages, glycolysis is up-regulated not only to provide ATP in a faster way, but also to promote the TCA cycle for efficient generation of acetyl-CoA from citrate [98]. Consistent with this, the level of citric acid-transforming ACLY in activated macrophages increases rapidly [99]. In addition, the expression of CIC is also up-regulated in LPS-activated macrophages, and the inflammatory mediators are decreased when CIC activity is inhibited [100]. ACLY is an enzyme that directly acts on the downstream of CIC, but surprisingly, in LPS-induced macrophages, the upregulation of the ACLY gene is observed earlier than the activation of CIC, despite CIC providing a substrate for ACLY activity. A reasonable explanation is that the substrate of ACLY is cytoplasmic citric acid, which may in turn activate CIC as an inflammatory signal when cytoplasmic citric acid is exhausted. ACLY plays a crucial role in the inflammatory response of macrophages and the production of inflammatory mediators, including PGE2, ROS, and NO. Inflammatory stimuli such as TNF-α, IFN-γ or LPS activate the ACLY through NF-κB and STAT signals during the early stage. ACLY provides acetyl-CoA as the raw material for PGE2 synthesis, which subsequently forms arachidonic acid under the action of desaturases and elongases, serving as a precursor to PGE2. In addition, apart from facilitating the generation of acetyl-CoA, ACLY also facilitates the synthesis of oxaloacetic acid. Intracellular NADPH and H^+^ are produced during the reduction of malic acid to pyruvate. Therefore, ACLY can also provide the NADPH necessary for ROS and NO production that induces inflammatory responses of macrophages [99]. This suggests that ACLY plays a pivotal role in mediating early-stage inflammation, thereby potentially serving as a novel biomarker for predicting inflammatory status and representing a promising therapeutic target for inflammatory diseases.

### ACC

ACC catalyzes the ATP-dependent carboxylation of acetyl-CoA and promotes the conversion of acetyl-CoA to malonyl-CoA, which is the rate-limiting step in fatty acid biosynthesis. Eukaryotic acetyl-CoA carboxylase is a large homodimeric multienzyme complex. In humans, there are two isozymes of ACC: cytoplasmic ACC1 involved in metabolic processes and mitochondrial membrane-anchored ACC2 responsible for regulating fatty acid oxidation [101]. ACC1 is a cytoplasmic enzyme that primarily serves as a substrate in the adipogenic pathway to generate malonyl-CoA, whereas locally produced malonyl-CoA by ACC2 regulates fatty acid β-oxidation by inhibiting acylcarnitine transport into mitochondria via carnitine palmitoyltransferase 1 (CPT1). Scott et al. demonstrated that the absence of ACC leads to an upregulation of glycolysis in macrophages, impairing their ability to enhance glucose utilization and preventing TLR-induced lipid accumulation. Deletion of the ACC gene attenuates the transcriptional activation of pro-inflammatory genes, thereby compromising the secretion of pro-inflammatory cytokines by macrophages such as IL-6 and IL-1β, as well as pathogen clearance and phagocytosis, suggesting a crucial role for ACC in inflammatory activation and metabolic reprogramming of immune cells [102], which may affect ALI. Previous studies have indicated that stearoyl lysophosphatidylcholine (LPC) exerts a protective effect against lethal experimental sepsis by inhibiting the LPS-induced extracellular release of high-mobility group box (HMGB1). In mice lungs, administration of stearoyl-LPC results in increased phosphorylation of ACC, a downstream target of activated AMPK, and concomitantly reduces HMGB1 levels in bronchoalveolar lavage fluids following LPS exposure [103]. Moreover, metformin, an AMPK activator, exhibits potential in mitigating lung damage in an animal model of ARDS, while activation of the AMPK/ACC signaling pathway enhances mitochondrial function and reduces TGF-β-induced fibrosis, apoptosis, and inflammation markers in lung epithelial cells [104]. Thus, ACC can be regarded as a promising target for lung protection and immunoregulation.

### FASN

Fatty acid synthase is a homodimeric protein, with each monomer consisting of six distinct enzyme domains that collaborate in the synthesis of 16-carbon saturated fatty acid palmitates from acetyl-CoA and malonyl-CoA units, thereby facilitating de novo fatty acid synthesis. The ketoacyl domain of FASN catalyzes the synthesis of acetoacetyl-CoA from malonyl-CoA and acetyl-CoA, which is an essential step in cholesterol biosynthesis. Previous studies have demonstrated a connection between FASN and cholesterol synthesis. During LPS-induced inflammation response, FASN changes cellular cholesterol levels in a faster way, which is critical during inflammation. As cholesterol serves as a vital constituent of the cell membrane and plays a pivotal role in macrophage activation during the inflammatory response, the anti-inflammatory effect of inhibiting FASN has nothing to do with the whole enzyme and its ability to produce palmitate, but the domain of ketoacyl synthetase [105]. FASN plays an important role in the transduction of inflammatory signals and regulation of lipid biosynthesis in macrophages in endotoxin response, and the ketoacyl synthase domain of FASN is indispensable for macrophage activation. The expression of FASN in the lung is significantly high, mainly in AECIIs [106]. Specific knockout of AECIIs FASN is associated with exacerbated lung injury and increased alveolar permeability [107], resulting in compromised metabolic potential under stress conditions and reduced maximum respiration and reserve capacity. Furthermore, a high-fat diet can down-regulate the expression of FASN in pulmonary tissues, which is associated with impaired mitochondrial metabolic capacity under stress and increased susceptibility to acute lung injury, besides, compromised lipid production further exacerbates cellular apoptosis [107].

The expression of FASN in pulmonary endothelial cells is up-regulated in LPS-induced ALI mice [108]. Endothelial dysfunction occurs in the early stage of acute lung injury and plays a pivotal role in its pathogenesis. The integrity of the microvascular barrier relies on adherens junction protein located in cell–cell boundaries, while FASN is a key mediator of functional alterations in pulmonary endothelial cells under endotoxin-induced metabolic stress. By activating the p38 MAPK/NLRP3 signaling cascade, FASN inhibits VE-cadherin expression and promotes pulmonary vascular leakage in response to endotoxin stress [108], while inhibition of FASN facilitated the repairment of pulmonary endothelial cell barrier integrity under metabolic stress, as evidenced by the upregulation of VE-cadherin expression, enhanced trans endothelial electrical resistance (TEER), and reduced permeability following endotoxin stimulation. The palmitoylation process of endothelial nitric oxide synthase relies on FASN activity, which plays a maintaining endothelial function by facilitating eNOS targeting to the plasma membrane [109]. Another research has reported that miR-335-5p targets and suppresses FASN upstream, leading to activation of the AMPK/ULK1 signaling pathway and subsequent enhancement of autophagy. This cascade ultimately mitigates the inflammatory response in septic mice [110].

### Desaturases and elongases

Stearoyl-CoA desaturase 1 is a lipase that converts saturated fatty acids (SFAs) into monounsaturated fatty acids (MUFAs), serving as a key regulator of regulating fatty acid metabolism. Cyclic adenosine monophosphate response element binding protein 1 (Creb1) is a transcription factor that mediates cyclic adenosine monophosphate (cAMP) signaling in many tissues. Creb1 plays an important role in the induction and maintenance of SCD1 in developing fetal rat type II lung epithelial cells [111]. It has been reported that SCD1 exerts robust regulatory control over cellular inflammation and stress response across diverse cell types and disease conditions, including hepatocytes, islet β cells, macrophages, adipocytes, and endothelial cells. Nevertheless, the role of SCD1 in lung injury remains unreported, thus further investigations are warranted to elucidate its physiological and pathological implications in ALI.

Linoleic acid (LA) is metabolized by desaturase, including FADS1,FADS2, and elongase of very long-chain fatty acids 2 (ELOVL2) [112]. LA was first converted to γ-linolenic acid (GLA) under the action of FADS2, while the subsequent conversion of GLA to dihommo-γ-linolenic acid (DGLA) is catalyzed by both FADS1 and ELOVL2. Ultimately, DGLA undergoes further transformation into arachidonic acid (AA) [113]. Serum LA are found to be elevated during the progression of ALI. Previous studies have demonstrated that LPS and LPS-induced cytokines, such as IL-6 and TNFα, exert inhibitory effects on desaturase expression and activity, thereby impeding the conversion of LA to AA. Liu et al. find that protectin conjugates in tissue regeneration 1 (PCTR1), a recently identified member of specialized pro-resolving mediators (SPMs), can mitigate LPS-induced acute lung injury by facilitating the conversion of serum LA to intrahepatic AA through the upregulation of FADS1, FADS2, and ELOVL2 expression while reducing phospholipaseA2 (PLA2) expression [114], indicating that desaturases may play an important role in the metabolism of pro-inflammatory fatty acids. In fact, the involvement of desaturases and elongases in fatty acid metabolism is much more complex than that mentioned above. Cis-linoleic acid and α-linolenic acid (ALA) are desaturated and elongated by the same group of enzymes to form their respective long-chain polyunsaturated fatty acids (LC-PUFAs), such as GLA, DGLA, AA, eicosapentaenoic acid (EPA), and docosahexaenoic acid (DHA). DGLA is the precursor of series 1 prostaglandins (PGs), while AA is the precursor of series 2 PGs, thromboxanes (TXs), and series 4 leukotrienes (LTs). EPA serves as the precursor of series 5 PGs and TXs and series 5 LT [115]. Most PGs, TXs and LTs possess pro-inflammatory properties. However, it is noteworthy that AA also serves as the precursor for lipoxin A4 (LXA4), which exhibits potent anti-inflammatory effects. LXs is formed by platelets dependent on white blood cells to produce LTA4 through cross-cell biosynthesis, and platelet 12-lipoxygenase catalyzes the conversion of LTA4 into both LXA4 and LXB4. Hemolysin is a kind of anti-inflammatory bioactive lipids derived from EPA and DHA. In addition, DHA also forms precursors of another type of anti-inflammatory compounds called resolvins. Lipoxins, hemolysin and resolvins exert robust anti-inflammatory actions by inhibiting the production of free radicals, myeloperoxidase activity, secretion of IL-6, TNFα as well as the expression of high-mobility group protein 1 [116, 117]. Moreover, in the process of septic acute lung injury, the increase of TNFα will induce EFA-deficiency-like state, potentially altering the metabolism of essential amino acids and various polyunsaturated fatty acid derivatives [118]. Therefore, whether desaturases and elongases express pro-inflammatory or anti-inflammatory properties in ALI still needs further discussion and research (Table 1).

**Table 1:** In vivo experiments of fatty acid metabolic pathways involved in acute lung injury

Process of fatty acid metabolism	Target	Cell type	Consequence	Model	Refs.
FA uptake	FABP4	Lung epithelial cell	Inhibition of FABP4 suppresses ROS expression and reduces production of CXCL-8, TNFα, IL-6 and IL-1β	CLP-induced ALI	[29]
	FABP5	Macrophage	S-glutathionylation of FABP5 activates PPARβ/δ, and suppresses macrophage inflammation	LPS-induced ALI Hyperoxia-induced ALI	[35]
	FATP1	Macrophage	FATP1 regulates the production of inflammatory cytokines through ceramide and the JNK signaling pathway	LPS-induced ALI	[19]
	CD36	Macrophage	CD36 promotes macrophage M1 polarization by regulating CD14 associated with TLR4	LPS-induced ALI	[21]
		Lung endothelial cell	CD36 functions as a ligand for truncated oxidized phospholipids and mediates endothelial cell dysfunction	Tr-OxPLs–induced lung injury	[41]
	CAV-1	Macrophage	Knockdown of CAV-1 improve ALI by decreasing the expression levels of CD3 and F4/80, and activating autophagy by inhibiting AKT/mTOR and promoting the AMPK signaling pathway	LPS-induced ALI	[45]
	FFAR2	Macrophage	Up-regulated FFAR2 inactivates NLRP3 inflammasomes by regulating mitochondrial fission	CLP-induced ALI	[53]
		Lung epithelial cell	Overexpression of FFAR2 can ameliorate ALI and apoptosis through JNK/ELK1 signaling pathway	LPS-induced ALI	[54]
	GPR84	Neutrophil	GPR84 induces neutrophils to produce ROS by activating Lyn, AKT and ERK1/2 and the assembly of NADPH oxidase	LPS-induced ALI	[62]
FA oxidation	PGC-1α CPT1A MCAD LCAD	Lung epithelial cell	FAO is essential to AEC bioenergenesis and functional homeostasis, and FAO impairment-induced AEC dysfunction mediates ALI	LPS-induced ALI	[13]
	CPT1A	Lung endothelial cell	Enhancing FAO protects against hyperoxia-induced endothelial cell apoptosis and lung injury	Hyperoxia-induced ALI	[67]
FA synthesis	ACC	Macrophage	Activation of AMPK/ACC reduces extracellular release of HMGB1	LPS-induced ALI	[103]
		Lung epithelial cell	Activation of the AMPK/ACC signaling pathway enhances mitochondrial function and reduces TGF-β-induced fibrosis, apoptosis, and inflammation markers in lung	LPS-induced ALI	[104]
	FASN	Lung epithelial cell	Depletion of FASN results in altered mitochondrial bioenergetics and more severe lung injury	Hyperoxia-induced ALI	[107]
		Lung endothelial cell	FASN inhibits VE-cadherin expression and promotes pulmonary vascular leakage by activating the p38 MAPK/NLRP3	LPS-induced ALI	[108]

## The impact of free fatty acids and its derivatives on ALI

Free fatty acids are products of neutral fat metabolism and represent one of the main causes of lung injury. Notably, a significant elevation in serum FFAs has been observed in mice with lung injury [21]. However, the precise mechanism of lung injury caused by FFAs remains elusive. A recent study has proposed that FFAs can elicit the release of neutrophil extracellular traps (NETs) through NOX-dependent pathways as well as p38, ERK, and JNK signaling cascades. Furthermore, NETs induced by FFAs have been shown to promote dendritic cells (DCs) activation and differentiation into Th1 and Th17 cells, thereby playing a pivotal role in ALI [119], which offers a novel insight into the mechanism of lung injury induced by FFAs. In addition to the anabolism and catabolism pathways in the cytoplasm, some FAs can also undergo enzymatic conversion into substrates of lipid mediators, which exert their biological activities in tissue inflammation and organ damage.

### Eicosanoids

Eicosanoids play an important role in the regulation of pro-inflammatory and anti-inflammatory events during ALI. Omega-6 polyunsaturated fatty acids (ω-6PUFA), including arachidonic acid, represent the main polyunsaturated fatty acids in common Western diets and serve as the main pro-inflammatory eicosanoid in ALI. Omega-3 polyunsaturated fatty acids (ω-3PUFA) are essential fatty acids derived from fish oil, comprising EPA, DHA, and docosapentaenoic acid (DPA). They can be derived from ω-6PUFA desaturation and chain elongation, serving as alternative lipid precursors for the cyclooxygenase and lipoxygenase pathways [120]. The release of pro-inflammatory cytokines is related to the ω-3/ω-6 ratio in alveolar cell membranes. In conditions of high DHA supply, there is an increase in the proportion of ω-3PUFA and a decrease in the release of pro-inflammatory cytokines. Conversely, under high AA supply, both the proportion of ω-6PUFA and the release of pro-inflammatory cytokines increase. These findings provide a biochemical rationale for recommending a shift from omega-6 to ω-3 PUFA supplementation in nutritional support for patients with ALI [121]. Furthermore, fish oil-based lipid preparations not only modulate the ω-3/ω-6 ratio and suppress the production of pro-inflammatory cytokines but also attenuate monocyte rolling and adhesion to endothelial cells, thereby mitigating neutrophil recruitment to the bronchoalveolar chamber [122]. Interestingly, despite the well-established pro-inflammatory role of AA in acute lung injury, it also exerts a protective effect by preventing SFA-induced TLR4 from forming complexes with its accessory proteins. Specifically, AA directly binds to myeloid differentiation factor 2 (MD2), a co-receptor for TLR4, and impedes the activation of TLR4-mediated pro-inflammatory signaling pathway induced by SFA. Thus, through binding to MD2, AA attenuates LPS-induced macrophage inflammation and ALI in mice [123]. Moreover, epoxyeicosatrienoic acids (EETs) derived from AA also exhibits anti-inflammatory properties. EETs exert their therapeutic effects in the treatment of ALI by inhibiting calcium overload and ROS generation in macrophages and suppressing the activation of NLRP3 inflammasomes [124].

The early stage of ALI is characterized by neutrophilic inflammation, increased permeability, and intravascular and intraalveolar fibrin deposition. Eicosanoid derivatives of prethrombotic fatty acid cyclooxygenase (such as thromboxane A2) and 5-lipoxygenase (such as leukotriene B4) are mediators of these processes [125, 126]. The composition of membrane phospholipids determines the type and inflammatory activity of eicosanoids released during inflammation. Omega-6 fatty acids are responsible for generating highly active and pro-inflammatory PGE2 as well as series 4 leukotrienes, whereas omega-3 fatty acids facilitate the production of less active and potentially anti-inflammatory PGE3 along with series 5 leukotrienes [122]. A diet containing specific fat components may affect inflammatory and immune events. The beneficial effects of ω-3 fatty acids have been confirmed in experimental models of ALI [127, 128]. In patients with ALI or sepsis, enteral supplementation of ω-3 fatty acids and antioxidants reduces ventilator time, improves oxygenation index, and shortens stay in intensive care unit. However, with the popularity of enteral supplementation of ω-3 fatty acids in severe ALI/ARDS, a growing number of subsequent studies including a large multicenter trial conducted by ARDSNet investigate the effects of the enteral supplementation on ARDS patients and showed no beneficial effect [129, 130]. The aforementioned study was even prematurely terminated due to its lack of efficacy, and the result showed a higher occurrence of complications among patients supplemented ω-3 fatty acids. Due to the inconsistency of data on the use of ω-3 fatty acids in ALI/ARDS patients, there has been an intense scientific debate over the past decade, resulting in a dearth of more consistent recommendations.

Recently, a research team has synthesized ω-3 polyunsaturated fatty acid-alkanolamine (PUFA-AA) derivatives, which exhibit significant inhibitory effects on the expression of IL-6, TNF-α, and IL-1β induced by LPS and effectively mitigate the inflammatory response in LPS-induced ALI mouse. The anti-inflammatory mechanism of PUFA-AA is mediated through the NF-κB signaling pathway in a Nur77-dependent manner. These findings suggest that PUFA-AA derivatives hold promise as novel for potential therapeutic interventions against inflammatory diseases [131]. Besides, ALA is a plant-derived ω-3 fatty acid that possesses anti-inflammatory properties. Recent studies have demonstrated that pretreatment with ALA can ameliorate LPS-induced ALI by not only reducing the release of IL-1β cytokines from apoptosis cells but also inhibiting the activation of Pyrin inflammasome-driven macrophage sepsis [132, 133], offering a potential therapeutic option for managing ALI/ARDS through enteral nutrition.

### Specialized proresolving mediators

There is a growing recognition of that the regression of inflammation is actually a positive process. It has been found that a series of precursor mediators of biosynthesis in the process of acute inflammation, namely specialized proresolving mediators (SPMs), which are endogenous lipid metabolites derived from LC-PUFAs and serve as lipid regulators facilitating the resolution of acute inflammation. SPMs actively participate in all stages of inflammation regression, making their restoration crucial for achieving tissue homeostasis [134, 135]. The presence of SPMs helps to prevent the recruitment of granulocytes and facilitate macrophage-mediated clearance of apoptotic neutrophils and fragments, thereby promoting tissue homeostasis following injury. In addition, other immune cells, such as eosinophils, natural killer cells, and lymphocytes, are also affected by SPMs [136]. Insufficient levels of SPMs may contribute to the transition from acute to chronic inflammation. SPMs are enzymatically derived from long-chain PUFA such as AA, EPA and DHA, which are usually classified as lipoxins (LXs), E-series resolvins, D-series resolvins, protectins, and maresins [137].

Aspirin-triggered 15-epi-lipoxin A_4_ (15-epi-LXA_4_) and 17-epi-resolvin D1 (17-epi-RvD1) through the receptor ALX/FPR2 restore impaired phagocytosis and redirect human polymorphonuclear neutrophil granulocytes (PMNs) to apoptosis. Treatment of mice with 15-epi-LXA4 or 17-epi-RvD1 at the peak of inflammation enhances bacterial clearance, attenuates PMN accumulation, and promotes PMN apoptosis and efferocytosis, thereby facilitating resolution of E. coli- induced lung injury [138]. Moreover, treating septic mouse with IFN-β can enhance the production of 15-epi-LXA4 and RvD1 in the lungs, thus accelerating the resolution of pulmonary inflammation as mentioned above [139], suggesting that the lipid-mediated ALX/FPR2-centered proresolving pathway initiated by IFNβ has great potential in the treatment of ALI.

During ARDS, the increased permeability of the barrier between alveolar endothelial cells and epithelial cells leads to pulmonary edema and impaired gas exchange, while excessive neutrophil accumulation causes lung tissue damage. SPMs ameliorate ALI/ARDS mainly by modulating alveolar fluid clearance. In ALI rat model, LXA4 alleviates pulmonary edema by activating alveolar epithelial sodium channels to facilitate alveolar fluid clearance and suppressing the secretion of pro-inflammatory mediators such as TNF-α and IL-1β [140, 141]. Furthermore, both Rvd1 and MaR1 have also been shown to enhance alveolar fluid clearance in ALI animal models by modulating alveolar epithelial sodium channels and PI3K signaling pathways activated by Na, K-ATPase [142, 143].

In summary, preclinical models provide grounds for optimism that SPMs may prove to be a valuable tool in improving the prognosis of pathogenic infections, lung diseases and other inflammatory conditions.

### Nitrated fatty acid

Nitrated fatty acids (NFAs) are endogenous substances generated through the non-enzymatic reaction between nitric oxide (NO) and unsaturated fatty acids. NFAs suppress the release of IFN1 by inhibiting palmitoylation and signal transduction of stimulator of interferon genes (STING), thereby attenuating inflammation. Similarly, studies have shown that nitrate oleate exerts beneficial effects in preventing pulmonary inflammation induced by sepsis through inhibition of 5-lipoxygenase product generation, such as LTB4, 5-HETE, and 12-HETE [144]. NFAs can also inhibit the production of ROS in LPS-induced lung injury by down-regulating the expression of ROS-producing enzymes. In hyperoxia-induced lung injury, ROS production leads to a depletion of antioxidant capacity in the lungs, resulting in oxidative damage to DNA, lipids, and proteins. Specifically, NFAs exhibit antioxidant properties through its stimulation of the transcription factor Nrf2 via alkylating its inhibitor Keap1, thereby activating the antioxidant signaling pathway [145]. Recent research has demonstrated that NFAs can also mitigate oxidative stress-induced ALI by modulating heme oxygenase-1 (HO-1) and downstream target NQO1 regulated by Nrf2 [146]. These findings collectively suggest that NFAs represent an effective therapeutic approach for protecting against lung injuries with minimal side effects.

### Fatty acid ethanolamides

Fatty acid ethanolamides (FAEs) are endogenous lipids that exert anti-inflammatory property and play a crucial role in the modulation of many physiological functions [147]. Fatty acid amide hydrolase (FAAH) is a transmembrane protein responsible for the hydrolysis of the endocannabinoid N-arachidonoylethanolamide (AEA) and other related amidated signaling lipids, such as N-oleoylethanolamide (OEA), linoleoylethanolamide (LEA), and palmitoylethanolamide (PEA) [148]. Inhibition of FAAH exhibits a protective role in the development of ALI by suppressing the activation of NF-κB pathway as well as the expression of pro-inflammatory cytokines in a dose-dependent manner [149]. N-acylethanolamine acid amidase (NAAA) is a cysteine hydrolase generally expressed in immune cells, which is also responsible for the hydrolysis of FAEs. NAAA gene blockage exhibits a discernible anti-inflammatory effect on LPS-induced ALI, which resulting in elevated levels of PEA and AEA in bone marrow and macrophages, as well as an increased level of AEA in the lung [150]. Carmofur, a dual inhibitor of FAAH and NAAA, has been validated to exert anti-inflammatory effects in both raw264.7 macrophages and an LPS-induced ALI mouse model [151]. Furthermore, the administration of an ultramicronized preparation of PEA attenuates LPS-induced ALI in mice by inhibiting the activation of B-cells inhibitor alpha (IκBα) and NF-κB pathways, as well as reducing the expression of extracellular signal-regulated kinase 1/2 (ERK1/2), c-Jun N-terminal kinase (JNK) and p38 mitogen-activated protein kinase (p38/MAPK) expression. These findings suggest that PEA may serve as a potential adjunctive anti-inflammatory agent for the treatment of lung injury through modulation of the p38/NF-κB pathway [152].

### Short-chain fatty acids

Short-chain fatty acids (SCFAs) are a series of organic fatty acids with no more than six carbon atoms produced by symbiotic intestinal bacteria through anaerobic fermentation of dietary fiber and starch, including acetic acid, propionic acid, butyric acid, isobutyric acid, valeric acid and isovaleric acid. The production of SCFAs encompasses two pathways, the exogenous synthesis pathway mediated by intestinal bacteria and the endogenous production pathway involving host cell fatty acid oxidation [153]. The pivotal role of intestinal microbiota in governing gut homeostasis and contributing to the pathogenesis of severe acute pancreatitis-associated lung injury (PALI) has been widely recognized. In the systemic circulation and surrounding tissues of the host, only a fraction of SCFAs, such as acetate, propionic acid, and butyric acid, are absorbed and transported to the lungs [154]. However, during severe acute pancreatitis, PAMPs and SCFAs produced by intestinal microbiota can directly impact lung injury and inflammation through the gut-lung axis. Current studies have demonstrated the involvement of SCFAs in regulating alveolar macrophage polarization, immune cell migration, exosome production in lung tissue which promotes intercellular communication, and modulation of NETs formation as well as activation of damage-associated molecular patterns (DAMPs), thereby contributing to the development of PALI [155]. SCFAs can down-regulate the expression of pro-inflammatory cytokines including IL-1β, IL-6, TNFα, IFNγ and IL-8, while up-regulating the expression of anti-inflammatory cytokines IL-10 and TGFβ, thus inhibiting DAMP signal transduction in intestinal and lung tissues. Besides, the elderly constitutes a crucial patient population in ALI, and there is a chronic pro-inflammatory environment in the elderly, which is closely associated with the poor prognosis of ALI. Studies have confirmed a positive correlation between distinct intestinal microbiota and pulmonary inflammation during aging, suggesting that the gut-lung axis exerts an influence on inflammation-aging processes. However, supplementation of SCFAs significantly attenuates augmented inflammatory signals in ALI among aged mice, thereby mitigating inflammatory damage, oxidative stress, metabolic alterations, and myeloid cell activation [156]. In fact, the exact therapeutic efficacy of sodium butyrate (SB) and valproic acid (VPA) in ALI has been confirmed, as SB significantly inhibits the production of pro-inflammatory cytokines, the release of HMGB1 and the activation of NF-κB [157], while VPA can reduce the production of neutrophil chemokine-1 (CINC-1) to ameliorate hemorrhage-induced ALI [158]. Interestingly, VPA is also a broad-spectrum HDAC inhibitor, which shows great therapeutic potential in many inflammatory and autoimmune diseases. VPA can prevent the loss of histone acetylation induced by ischemia–reperfusion, thereby reducing pulmonary edema, suppressing the production of inflammatory cytokines, ROS, mitigating NF-κB signal transduction and apoptosis by increasing the activity of HO-1 [159]. In a word, SCFAs plays an extremely important role in maintaining intestinal barrier function, inhibiting bacterial migration, regulating immunity, and lung pathophysiology. The strong epigenetic regulation of SCFAs, complex network regulation mechanism, and possible synergism with other functional metabolites are worthy of further investigation.

## Obesity reprograms fatty acid metabolism and exacerbates ALI

According to the statistics of the World Health Organization, there has been a nearly threefold increase in the global prevalence of obesity since 1975. Currently, more than 1 billion individuals worldwide are affected by obesity, encompassing 650 million adults, 340 million adolescents, and 39 million children. Within intensive care units, obese patients represent a distinct population with approximately 20% of ICU patients being classified as obese [160]. Furthermore, these patients face an elevated risk for developing ARDS. While it is widely acknowledged that obesity serves as a significant risk factor for ALI/ARDS, the precise mechanisms through which obesity exacerbates the inflammatory response in ALI remain incompletely understood.

In obese individuals, the increase of circulating FFAs caused by metabolic overload has been shown to affect immune cells and chronic inflammatory processes. After ventilator-induced lung injury, BALF FFAs in mice increased, and further increased in mice with high-fat diet, which may be an indicator of elevated alveolar permeability [107]. As has been mentioned above, certain fatty acids, such as ω-3PUFA or MUFA, have the potential to mitigate inflammation through diverse mechanisms, while SFA may exacerbate the pro-inflammatory response. In humans, adipose tissue can be categorized into subcutaneous adipose tissue (SAT) and visceral adipose tissue (VAT). Recently, the presence of VAT has also been identified in the lungs, where it is stored within lipofibroblasts, these specialized cells play a pivotal role in regulating alveolar lipid homeostasis, facilitating pulmonary surfactant production, and enhancing oxygen uptake [161]. During the period of overnutrition, excess nutrients are stored in adipose tissue in the form of lipids, leading to obesity, while the surplus FFAs are deposited as ectopic fat in various organs, inducing excessive production of ROS and pro-inflammatory effects [162]. The possible mechanism is that after TLR4 in resident adipocytes and macrophages is activated, FAs activate NF-κB and P38MAPK signals through downstream pathways mediated by MyD88 and TRIF, thereby enhancing endoplasmic reticulum stress, ROS generation, and promoting the secretion of pro-inflammatory cytokines [163].

Obesity leads to an upregulation of major proteins associated with fatty acid uptake and transport. CD36 facilitates the capture and transfer of FAs to LDs, thereby effectively coupling FA uptake through CD36-mediated endocytosis. CD36 is widely overexpressed in obese animal models and humans [164], which may be attributed to its involvement as a lipoprotein scavenger receptor in cholesterol metabolism. Obesity induces up-regulated CD36 expression in preadipocytes. Dysregulated CD36 levels in preadipocytes can disrupt lysosomal calcium homeostasis and impair lysosomal function, thus exacerbating adipose tissue inflammation and contributing to the inflammatory response associated with ALI. Consequently, targeting lysosomal calcium balance represents a novel therapeutic strategy for mitigating obesity-induced inflammation [165]. In addition, the serum FABP4 level of obese mice increases significantly [166], suggesting that obesity may further enhance the role of FABP4 in promoting the release of pro-inflammatory molecules such as TNFα and IL-6.

Studies have shown that triglycerides accumulated in obese individuals are mainly originates from de novo fatty acid synthesis rather than other lipids, as obese mice exhibit elevated activity levels of ACLY, FASN, and malic enzyme, compared to lean mice [167]. ACC serves as the first rate-limiting enzyme in de novo fatty acid synthesis and plays a crucial role in both fatty acid production and oxidation, and AMPK is recognized as a classic phosphorylated kinase of mammalian ACC. Increased dietary fat intake can up-regulate ACC expression while inhibiting the AMPK/ACC pathway phosphorylation, thereby attenuating the inhibitory effect of AMPK on ACC activity and suppressing the anti-inflammatory properties of AMPK [168]. Moreover, FASN is up-regulated in pulmonary endothelial cells of LPS-induced ALI mice, which is further enhanced by obesity. Inhibition of FASN in obese mice effectively restores the loss of VE-cadherin in pulmonary endothelial cells and exerted a beneficial effect on reducing pulmonary vascular leakage during ALI [108]. Conversely, high-fat diet results in the down-regulation of FASN expression in AECs, which is associated with impaired mitochondrial metabolic capacity under stress and more severe lung injury [107].

Obesity can induce a reduction in FAO, leading to intracellular lipid accumulation and rendering cells more susceptible to dysfunction [169]. Ceramide has been shown to be involved in inflammation, apoptosis, ROS production, endoplasmic reticulum stress, and autophagy through diverse mechanisms. Obesity creates an ideal milieu for ceramide synthesis due to the elevated levels of SFAs and inflammatory state [170]. A special ceramide, C16:0, has been identified as a key negative regulator of FAO and insulin sensitivity in obesity [171]. The crosstalk linking obesity, ceramide, and FAO might be that obesity primarily increases SFAs and CerS6, resulting in the accumulation of C16:0, which subsequently lead to dysfunction of the electron transport chain and the production of ROS. Consequently, ROS inactivates CPT1 and suppresses FAO, thus promoting intracellular lipid accumulation [169]. Down-regulated FAO weakens the ability of obese ALI patients to maintain intracellular metabolic homeostasis during inflammatory shock and oxidative stress. In addition, as mentioned above, the activation of NMDAR impairs FAO by reducing the phosphorylation and activity of PPARα through ELK1/2 pathway, and NMDAR is overactivated in high-fat diet-induced obese mice [64], suggesting that the FAO of obese patients may be further damaged during ALI, which in turn aggravates the lung injury.

Obesity is a state of excessive fat storage and overmuch LDs in adipose tissue, which is usually accompanied by fat deposition and excessive LDs in non-adipose tissue, resulting in lipotoxicity and tissue dysfunction [172]. In addition, obesity is also associated with disordered lipolysis and chronic inflammation. Previously, caspase recruitment domain-containing protein 9 (CARD9) has been identified as a potential contributor to obesity-related abnormalities. Activation of the CARD9 signaling complex by PKCδ leads to the upregulation of transcription factors p38 MAPK and NF-κB, as well as the production of pro-inflammatory cytokines such as IL-6, TNFα, and IL-1β [173]. Besides, obesity induced by high-fat diet is associated with an imbalance in low-density lipoprotein lipolysis in macrophages, leading to the activation of DAG-PKCδ signaling, CARD9-dependent inflammation, and impaired fat phagocytosis. In turn, impaired fat phagocytosis may exacerbate imbalance between excessive lipid accumulation and fat decomposition, thus maintaining a potential positive feedback loop of obesity-related inflammatory states [174], which may contribute to the aggravation of acute lung injury in obese patients. Interestingly, a selective overexpression of DGAT1 in mice macrophages and adipocytes exhibits a higher propensity for obesity, however, up-regulated DGAT1-dependent triacylglycerol storage ameliorates metabolic complications arising from obesity, including inflammatory macrophage activation, macrophage aggregation in white adipose tissue, systemic inflammation, and insulin resistance. These findings suggest that enhancing the capacity of macrophages to store triacylglycerol may mitigate inflammation triggered by SFAs [175].

**Fig. 2:**
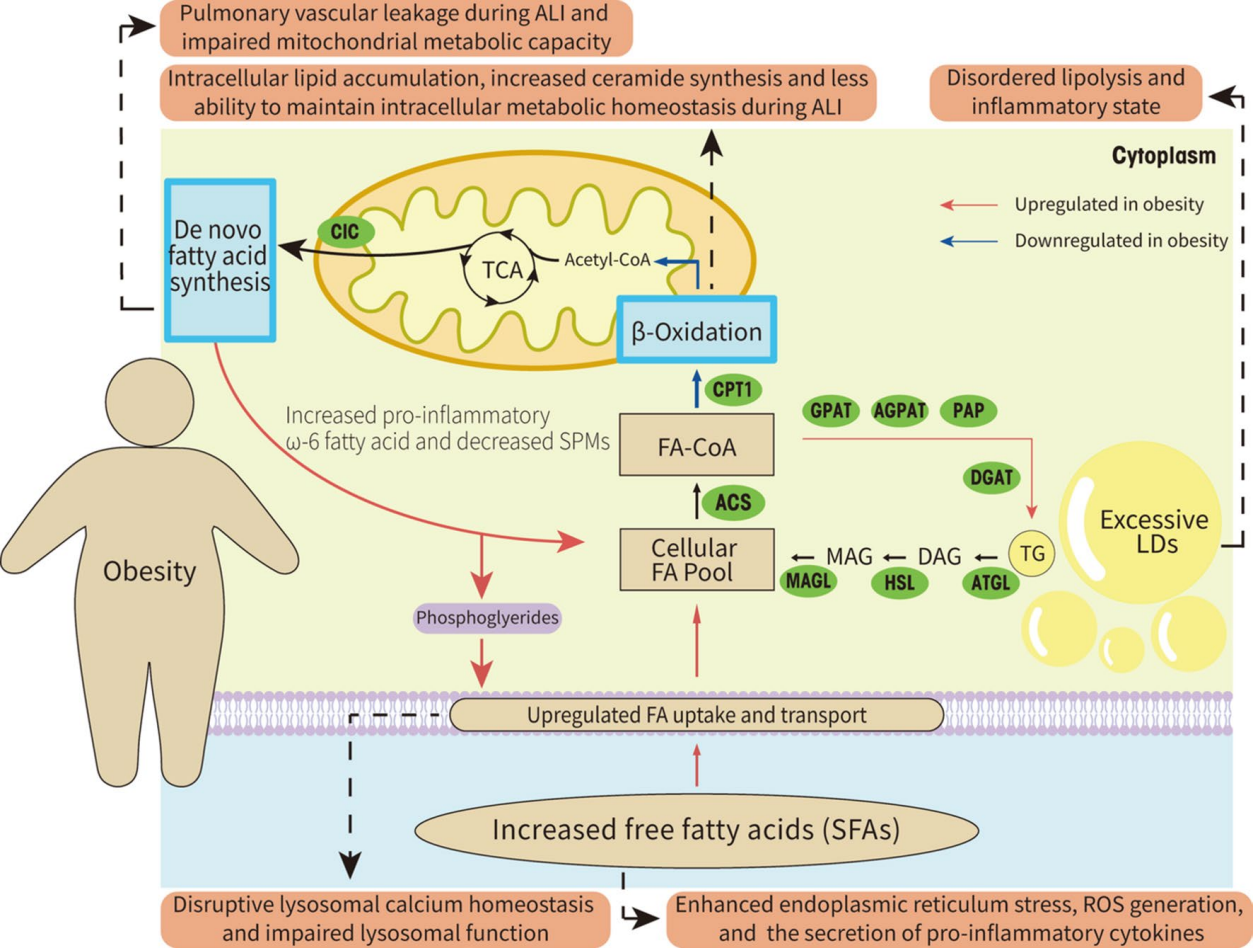
Obesity reprograms fatty acid metabolism and exacerbates lung injury. In obese individuals, metabolic overload causes the increase of circulating FFAs, as well as leading to an upregulation of major proteins associated with fatty acid uptake and transport. Besides, obesity is a state of excessive LD accumulation, disordered lipolysis and chronic inflammation. Triglycerides accumulated in obese individuals are mainly originates from de novo fatty acid synthesis rather than other lipids. Furthermore, obesity leads to changes in fatty acid composition and their derivatives, including increased ω-6 fatty acids and decreased SPMs. All these metabolic alterations may further exacerbate ALI

Another group of factors that control the susceptibility of obesity to lung injury and inflammation are LC-PUFAs and their derivative oxylipins. These oxylipins, which mainly include prostaglandins and leukotrienes, are synthesized by ω-6 fatty acid arachidonic acid and play a key role in initiating inflammation [176]. In addition, oxylipins also include SPMs and their metabolic intermediates, which promote the decomposition of inflammation and restore homeostasis of damaged tissue. Obesity disrupts the homeostasis of PUFA and their oxylipins in pulmonary tissue, thereby exacerbating lung inflammation through lipid oxidation imbalance. In individuals with obesity, LTB4 content in leukocytes exhibits a fourfold increase, while levels of EPA and DHA-derived oxylipins are significantly reduced [177]. Furthermore, the levels of SPMs and its intermediates or precursors are significantly reduced in obese individuals [178]. Alterations in these metabolites may aggravate ALI damage to some extent. The association between decreased SPM levels and poor prognosis of ALI has been confirmed in obese patients infected by SARS-CoV-2 [179]. Interestingly, obesity does exacerbate the inflammation of ALI, however, prior to lung injury induced by external factors, obesity has modulated the metabolism of PUFAs in lungs. The administration of a high-fat diet increases the relative abundance of triglycerides containing PUFAs and the concentration of PUFA derived oxylipins in lungs, thereby up-regulating pathways associated with glycerol phospholipid metabolism and immunity, including a unique upregulation in B cell differentiation and signal transduction [180]. These findings are of great significance in understanding why obesity leads to a poor response to infectious and inflammatory challenges (Fig. 2).

## Conclusion

Fatty acid metabolism involves cellular fatty acid uptake and storage, transport to mitochondria, mitochondrial fatty acid oxidation and fatty acid synthesis. In a word, the fatty acid metabolic pathways play a crucial role in maintaining pulmonary homeostasis. With the emergence of immunometabolism as a field of study and the escalating burden of obesity-related diseases, researchers are progressively shifting their focus towards exploring the relationship between fatty acid metabolism and acute lung injury. During ALI, inflammation and oxidative stress lead to energy depletion, resulting in impaired fatty acid oxidation, increased expression of proteins involved in fatty acid uptake and transport, enhanced synthesis of fatty acids, and accumulation of LDs. These metabolic alterations represent a response to the challenge of ALI rather than active adaptive changes. Notably, reversing the expression of key enzymes involved in fatty acid metabolism can effectively mitigate the severity of ALI. Therefore, targeting fatty acid metabolism holds great promise for future research on lung protection. If patient’s fatty acid metabolic pathway can be regulated through dietary and pharmacological interventions in advance, enabling them to be in a state of readiness for stress and prepared for being hit by ALI, just as obesity keeps the body in a state of chronic low-degree inflammation. Pre-regulation could potentially mitigate the severity of ALI and reduce mortality. In conclusion, this paper presents a systematical review of the relationship between extensive fatty acid metabolic pathways and acute lung injury and summarizes recent advances in understanding the involvement of fatty acid metabolism-related pathways in ALI. However, the specific regulatory mechanisms are way too complex, necessitating further extensive and in-depth investigations in future studies.

## Data Availability

This review article did not look at any new data. Only results published in previous studies and identified in the reference list below were used.

## Abbreviations

ACC: Acetyl-CoA carboxylase
ACLY: ATP- Citrate lyase
AEC: Alveolar epithelial cell
ALI: Acute lung injury
AM: Alveolar macrophage
ARDS: Acute respiratory distress syndrome
ATGL: Adipose triglyceride lipase
CAV-1: Caveolin-1
CPT: Carnitine palmitoyltransferase
DHA: Docosahexaenoic acid
ELOVL: Elongase of very long-chain fatty acids
EPA: Eicosapentaenoic acid
FA: Fatty acid
FA-CoA: Fatty acyl-CoA
FABP: Fatty acid-binding proteins
FADS: Fatty acid desaturase
FAO: Fatty acid oxidation
FASN: Fatty acid synthase
FATP: Family of fatty acid transport protein
FFAR: Free fatty acid receptor
HSL: Hormone-sensitive lipase
IL: Interleukin
LD: Lipid droplet
MAGL: Monoacylglycerol lipase
PGE: Prostaglandin E2
PUFA: Polyunsaturated fatty acids
ROS: Reactive oxygen species
SCFA: Short-chain fatty acids
SFA: Saturated fatty acid
TLR: Toll-like receptors
TNF: Tumor necrosis factor
